# Editorial: Temporal Features in Resting State fMRI Data

**DOI:** 10.3389/fnins.2020.616513

**Published:** 2020-12-08

**Authors:** Nanyin Zhang, Xiaoping Hu

**Affiliations:** ^1^Pennsylvania State University, University Park, PA, United States; ^2^University of California, Riverside, Riverside, CA, United States

**Keywords:** resting-state fMRI, temporal features, functional connectivity (FC), brain network, neural circuit

It has become increasingly clear that functional connectivity derived from resting-state functional magnetic resonance imaging (rsfMRI) data is not stationary, but contains ample dynamic information (Hutchison et al., 2013). However, how to extract and quantify the temporal features in rsfMRI data, and how these features help us better understand the function of neural circuitries and networks in health and disease remain active Research Topics. This special issue includes a group of papers specifically investigating temporal features in rsfMRI data.

The utilization of temporal features in rsfMRI data has considerably enhanced our understanding of the neuropathophysiology and improved the diagnosis of brain disorders. Zhu et al. investigated the temporal variability on the connectivity profiles in Parkinson's disease (PD) using a sliding-window method. They found that PD patients exhibited greater temporal variability in both cortical and subcortical networks. Importantly, this dynamic connectivity measure was associated with the severity of clinical symptoms in patients (Zhu et al.). In another study, by integrating the strength and temporal variability of complex-network properties derived from effective connectivity networks in patients with post-traumatic stress disorder (PTSD), patients with PTSD with comorbid mild-traumatic brain injury (mTBI) and healthy controls, Rangaprakash et al. identified dysregulation of prefrontal regions over subcortical and visual regions in PTSD/mTBI. These patients displayed lower variability over time in all network properties, which can be an indicator of poorer flexibility. Importantly, authors show that network properties, including dynamic ones, of the prefrontal-subcortical pathway are not only significantly correlated to symptom severity and neurocognitive performance, but also provide predicative value in classifying these disorders (Rangaprakash et al.). Furthermore, using a novel high-order dynamic functional connectivity networks (D-FCNs), Zhao et al. achieved a classification accuracy of 83% for autism spectrum disorder (ASD). This method can overcome the problems of conventional sliding-window-based D-FCNs, such as lack of high-level interactions across regions and the temporal mismatching issue (Zhao et al.).

Temporal features in rsfMRI data are also tightly linked to physiology. For instance, converging evidence suggests that arousal may have profound effects on rsfMRI signals and connectivity dynamics. Gu et al. reviewed the relationship between rsfMRI and brain arousal. Authors also discussed the potential impact of arousal on spurious relationship between functional connectivity measures and physiology/behavior (Gu et al.). In addition, Wang et al. advanced the capacity of individual identification based on rsfMRI data by including temporal features in convolutional recurrent neural networks (ConvRNN). They demonstrate that ConvRNN can achieve a higher identification accuracy than conventional RNN (Wang et al.).

Methodology development continues to improve our capacity to extract temporal features of rsfMRI. Zhou et al. proposed a protocol designed to track dynamical whole-brain functional connectivity states, using signed community clustering with the optimized modularity by two-step procedures. This method makes it possible to track rapid dynamical change in functional connectivity (Zhou et al.). Chen et al. constructed brain networks using unwrapped fMRI phase image, instead of the magnitude of fMRI data conventionally used. The functional network connectivity matrix obtained based on phase fMRI was more sparsely distributed across the brain, with connections having more balanced positive/negative correlations. Their findings open a new avenue to understanding brain function connectivity (Chen et al.). Yang et al. went beyond the conventional method of regressing out motion parameters, and developed an automated convolutional neural network (CNN) model to obtain optimal motion regressors, which can better reduce motion-related artifacts.

Finally, we have a better understanding of temporal features in rsfMRI data. Guan et al. studied the linearity and stationarity of fMRI time series in individual resting-state brain networks, and found that different networks had distinct properties of non-stationarity and non-linearity. Nezafati et al. examined the complexity of regional brain activity measured by rsfMRI data using sample entropy. They found that the brain exhibited significant spatial heterogeneity in levels of entropy/complexity. In addition, the entropy of brain activity was also modulated by task performance (Nezafati et al.).

Taken together, the studies in this special issue demonstrate the importance of investigating temporal features in rsfMRI data, and exemplify the potential impact of applying these features to better understand brain function in health and disease.

## Author Contributions

NZ and XH wrote the editorial. Both authors contributed to the article and approved the submitted version.

## Conflict of Interest

The authors declare that the research was conducted in the absence of any commercial or financial relationships that could be construed as a potential conflict of interest.
